# Redo sphincteroplasty: are the results sustainable?

**DOI:** 10.1093/gastro/gov025

**Published:** 2015-06-29

**Authors:** Kwangdae Hong, Giovanna Dasilva, John T. Dollerschell, David Maron, Steven D. Wexner

**Affiliations:** Department of Colorectal Surgery, Cleveland Clinic Florida, Weston, FL, USA

**Keywords:** fecal incontinence, redo sphincteroplasty, long-term outcome

## Abstract

**Objective:** This study aimed to investigate the long-term outcomes of patients who undergo redo sphincteroplasty (RS).

**Methods:** Patients with fecal incontinence (FI) who underwent RS between November 1988 and December 2011 were retrospectively identified from a prospective database. A questionnaire and telephone survey assessed current Cleveland Clinic Fecal Incontinence Score (CCFFIS; best 0, worst 20) and Fecal Incontinence Quality of Life (FIQoL; best 4.1, worst 1) scale. Success was defined as no further continence surgery, no stoma and CCFFIS <9 at completion of follow-up. The Wilcoxon and Mann-Whitney U tests were used for comparing quantitative variables. Bivariate logistic regression analysis was done to identify predictive factors for success.

**Results:** Fifty-six (66.7%) of 84 patients who underwent RS were available for evaluation at a median follow-up of 74 (range: 12–283) months. The mean CCFFIS decreased from 16.5 ± 3.7 to 11.9 ± 6.6 (*P* < 0.001) at last follow-up. Twelve patients (21.4%) underwent further continence surgery for failed sphincteroplasty, three (5.4%) of whom had a permanent stoma. Eighteen patients (32.1%) had a CCFFIS <9 at the completion of follow-up, and 16 (28.6%) had long-term success. Twenty-four patients evaluated for FIQoL had a mean value of 2.6 (range: 1.0–4.1). Postoperative CCFFIS was correlated with FIQoL (Spearman’s correlation coefficient = −0.854, *P* < 0.001). Logistic regression analysis did not reveal any significant predictive variables for success of RS.

**Conclusion:** Based on our criteria for success, the long-term success rate for RS over a median of 74 months is poor.

## Introduction

Functional outcome of anal sphincter repair deteriorates over time [1,2]. The best surgical option for a failed repair is unknown. Current treatment options include biofeedback, injectable agents, radiofrequency treatment, redo sphincteroplasty (RS) or more invasive techniques such as artificial bowel sphincter or sacral nerve stimulation [3,4]. RS has been considered cost effective, has less morbidity, and therefore has been widely recommended for patients with persistent anal sphincter defect and a functional sphincter [5–7]. Whereas the long-term outcome of primary sphincteroplasty is well known [2], there is a paucity of data regarding the outcomes of a redo procedure. This study aimed to investigate the long-term outcomes of redo sphincteroplasty (RS).

## Patients and methods

After Institutional Review Board approval, all patients who underwent (RS) for fecal incontinence (FI) between November 1988 and December 2011 were identified from a prospective database, and their medical records were reviewed. Patients were selected for RS after failure of one or more previous anal sphincteroplasties. All patients had evidence of a sphincter defect on physical examination or endoanal ultrasonography, which was confirmed at the time of RS. The following parameters were recorded: age, gender, body mass index, history of diabetes mellitus, irritable bowel syndrome, length of incontinence symptoms, cause of FI, preoperative stoma, pre-RS Cleveland Clinic Florida Fecal Incontinence score (CCFFIS; best = 0, worst = 20) [8], type of external sphincter repair (overlapping repair vs imbrication) [9,10] and further continence surgery for FI. Preoperative manometric parameters, pudendal nerve terminal motor latencies and circumferential extent of the external sphincter defect on anal ultrasonography were also recorded. For patients with a colostomy, the preoperative CCFFIS was based on their historical level of incontinence prior to stoma construction. The length of follow-up for each individual patient was calculated from the day of RS to the last clinic visit using a postal or telephone questionnaire survey.

Postal questionnaire and telephone survey assessed the current CCFFIS, Fecal Incontinence Quality of Life (FIQoL) scale [11] and whether patients had further continence surgery for FI. In patients with a stoma at the completion of follow-up, the last CCFFIS and FIQoL were not calculated. Patients who did not have documented pre-RS CCFFIS in the medical record were excluded, as were patients with less than one year of follow-up.

There is currently no consensus regarding the most appropriate ‘success’ measure after sphincteroplasty. Rothbarth *et al.* definitely demonstrated that a CCFFIS of ≥9 indicates a significant impairment of QOL using validated scoring systems [12]. Hence, we defined clinical success as no additional surgery, no stoma and a CCFFIS <9 at the completion of follow-up.

## Statistical analysis

The Wilcoxon test and Mann-Whitney U test were used for comparing quantitative variables. The relationship with last CCFFIS and FIQoL was assessed using Spearman’s rho. The Fisher exact test was used for categorical variables. To identify the predictive factors for success of RS, bivariate logistic regression analysis was done. A *P* value < 0.05 was considered significant. Statistical evaluation was performed by use of the SPSS 15.0 (SPSS Inc., Chicago, IL, USA) for Windows.

## Results

Fifty-six (66.7%) of the 84 patients who underwent RS were available for follow-up. Among the 56 patients enrolled, 16 (28.6%) had prior repair at our institution, while 40 (71.4%) had their surgeries elsewhere. The patient’s preoperative clinical characteristics are listed in Table 1. The median follow-up duration was 74 (range: 12–283) months. Nine (16.1%) patients had two or more previous failed sphincteroplasties. The mean number of previous failed sphincteroplasties was 1.3 (range: 1–7). Only one patient received another form of treatment (SECCA procedure) before undergoing RS.

**Table 1.** Patient characteristics (*n* = 56)

Characteristics	Patients
Mean age, years	47.7 (20–73)
Female	55 (98.2)
Mean body mass index, kg/m2	27.1 (18.7–50)
Cause of fecal incontinence	
Obstetric injury	47 (83.9)
Anorectal surgery, trauma	5 (8.9)
Unknown	4 (7.1)
Mean length of symptoms, years	9.8 (0.5–39)
Comorbidities	
Diabetes mellitus	5 (8.9)
Irritable bowel syndrome	5 (8.9)
Preoperative stoma	3 (5.4)
Patients with two or more previous sphincter repair	9 (16.1)
Type of external sphincter repair	
Overlapping sphincter repair	53 (94.6)
Imbrication	3 (5.4)
Endoanal sonography^a^	
External anal sphincter (EAS) defect size, mean (range)	112 (80–180)
Internal anal sphincter (IAS) defect size, mean (range)	78 (0–180)
Pudendal nerve terminal motor latency^b^	
Normal	34 (77.3)
Unilateral abnormal	8 (18.2)
Bilateral abnormal	2 (4.5)
Anorectal manometry^c^	
Mean resting pressure, mmHg	29 (0–62)
Mean squeezing pressure, mmHg	31 (0–82)
Preoperative Cleveland Clinic Florida Fecal Incontinence Score (CCFFIS)	16.5 (8–20)

Data are number (percentage) or mean (range)

^a^Data available for 32 patients. ^b^Data available for 44 patients. ^c^Data available for 45 patients

**Figure 1.**
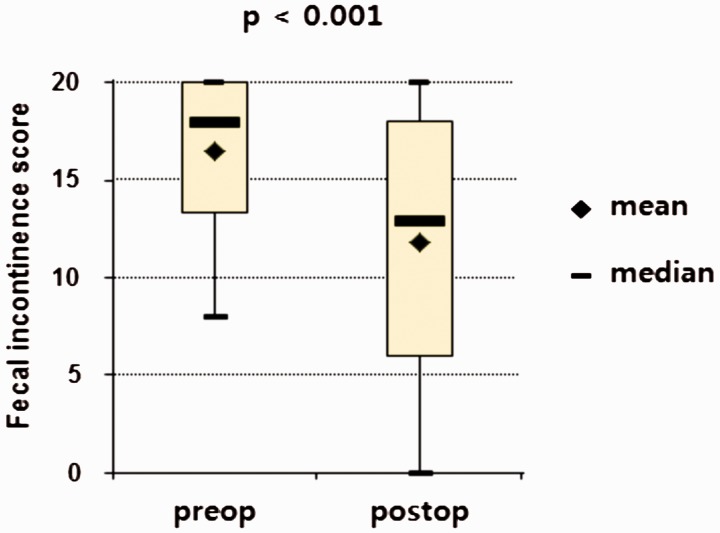
Preoperative and postoperative Cleveland Clinic Florida Fecal Incontinence Score (CCFFIS)

**Figure 2.**
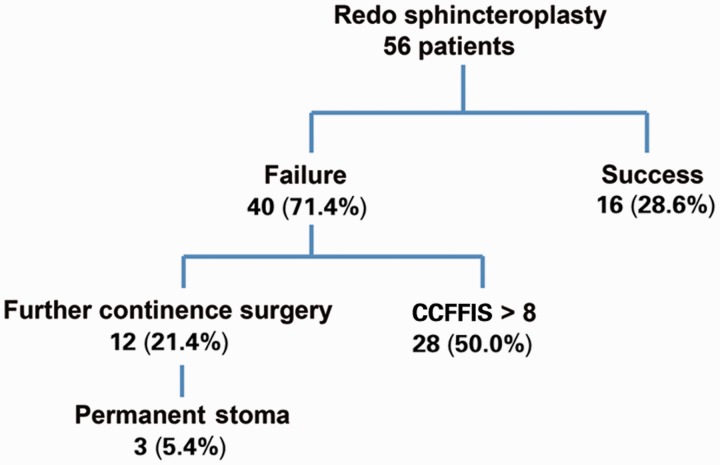
Outcomes of 56 patients after redo sphincteroplasty

The mean CCFFIS decreased from 16.5 (range: 8–20) to 11.8 (range: 0–20) after RS (*P* < 0.001) (Figure 1). Twelve patients (21.4%) underwent further continence surgery for failed RS: sacral nerve stimulation in two, artificial bowel sphincter in two, repeat RS in two, SECCA procedure in one, graciloplasty in three (one of whom had a subsequent stoma) and stoma construction in two; three patients (5.4%) had a stoma at the completion of follow-up. Eighteen patients (32.1%) had a CCFFIS <9 at the completion of follow-up. Based on our criteria for success, only 16 (28.6%) patients had long-term success (Figure 2).

Postoperative anal endosonography was available for 20 patients at a mean follow-up of 39 (range: 2–108) months; six patients (30%) had an external anal sphincter (EAS) defect, and 14 (70%) did not. Postoperative anorectal manometry data were available for 19 patients at a mean follow-up of 26 (range: 1–67) months. The mean resting and squeeze pressures were 31.5 (range: 0–52) mmHg and 28.5 (range: 0–60) mmHg, respectively. Preoperative and postoperative mean resting and squeezing anal pressures did not show any significant change (*P* = 0.890 and *P* = 0.749, respectively). In 16 patients, anal endosonography and manometry were performed at a mean of 33 (2–93) months, revealing that patients with an intact EAS had higher postoperative mean squeeze pressures compared with patients with an EAS defect (35.1 vs 14.2 mmHg; *P* = 0.029).

A total of 24 patients were evaluated with the FIQoL. The mean global value was 2.6 (range: 1.0–4.1) of the four scales (Table 2). Postoperative CCFFIS was significantly correlated with the four FIQoL scales (Table 3).

**Table 2.** Fecal Incontinence Quality of Life (FIQoL) Scale values (*n* = 24)

Scales of FIQoL	Mean value (range)
Life style	2.6 (1.0–4.1)
Coping/behavior	2.3 (1.0–4.0)
Depression/self-perception	3.0 (1.0–4.4)
Embarrassment	2.4 (1.0–4.0)
Global	2.6 (1.0–4.1)

**Table 3.** Relationship between Cleveland Clinic Florida Fecal Incontinence Score (CCFFIS) and Fecal Incontinence Quality of Life (FIQoL) Scale (*n* = 24)

Scales of FIQL	Spearman’s correlation coefficient with CCFFIS	*P* value
Life style	−0.778	<0.001
Coping/behavior	−0.859	<0.001
Depression/self-perception	−0.713	<0.001
Embarrassment	−0.875	<0.001
Global	−0.854	<0.001

Using a bivariate logistic regression analysis in the evaluation of a dependent variable of success, none of the clinical or physiological patient characteristics assessed in Table 1 was predictive for long-term outcome. In addition, postoperative manometry or anal endosonography, with a mean follow-up duration of 33 months, was not predictive for long-term outcome.

## Discussion

A prior publication about the long-term results of RS reported 62% clinical improvement with a median 60 months of follow-up [5]. These results are similar to the previous short-term results of RS [6,7]. However, in many studies, clinical success was determined by patient satisfaction. Recently, Glasgow *et al.* reported that patient QOL and satisfaction remain relatively high following anal sphincteroplasty, even though FI deteriorates over the long term [2]. This may be a result of psychological and behavioral adaptation by patients with a chronic condition. In addition, recent studies have revealed that good initial outcomes after sphincteroplasty deteriorate with time and that <50% of patients remain continent [1,9,10].

Rothbarth *et al.* reported that the CCFFIS is considered a reliable objective measurement, not only to assess FI but to also estimate the expected loss of QOL with a score of ≥9 [11]. Almost all patients with CCFFIS >9 were having stool seepage more than once a week, needed a diaper and felt restricted in their daily functioning. At the same time, the rate of further continence surgery can be an important objective measure of the long-term effectiveness of any surgical therapy for incontinence [1,12]. With these criteria in mind, we defined success as no further continence surgery, no stoma and a CCFFIS <9 at the completion of follow-up.

In our study, based on these specific criteria, the long-term success rate of RS over a median of 74 months was poor at 28.6%. Although we noticed significant improvement of the CCFFIS after RS, there were only 18 patients (32.1%) who had a CCFFIS <9 at the completion of follow up. Conversely, this lower rate of long-term success might indicate that the CCCFIS cut-off threshold value of 8 is too strict and consequently overestimates the severity of incontinence [13]. For similar reasons, Giordano *et al.* [6] defined poor outcome as CCFFIS >10, and Gallas *et al.* [14] chose a cutoff value of <30% reduction in the CCFFIS to define failure. Recently, the clinical success after SNS was assessed by the CCFFIS.

A major impediment in assessing the clinical effectiveness of surgery for FI is finding an appropriate measure of outcome. In order to compare treatment outcomes between other interventions or other centers, we need to use common objective measures. In this view, our criteria of clinical success seem to be reasonable and feasible.

A recent systematic review related to long-term outcome of sphincteroplasty reported that no patient or technical factors consistently translated to poor outcomes [2]. In our study, none of the patient clinical, physiologic or other features we studied were predictive for the long-term outcome for RS. Although a previous study from our institution suggested that patients with more than two previous sphincter repairs tended to have poorer outcome [6], our study (with a longer follow-up) did not show any relationship between the number of prior sphincter repairs and long-term outcome. Furthermore, postoperative endosonographic defect was not able to predict outcome. More conclusive studies are needed to determine any predictive factors.

RS has been considered appropriate for patients with persistent symptoms and a sphincter defect after failed sphincteroplasty [5–7]. In this selected case series, RS was recommended prior to pursuit of other treatment options. However, in view of poor long-term outcome, this approach may not be ideal. Further research is needed to identify the subgroup of patients who will potentially benefit from RS on a long-term basis.

This study has several limitations. It is a retrospective, non-controlled case series with a relatively small number of enrolled patients. Comparisons were made using two different types of data acquisition (direct interview preoperatively vs survey postoperatively), which can induce bias. In addition, data acquisition using questionnaires yields a modest return. In our determination of clinical success, we did not consider non-surgical treatments such as biofeedback before further continence surgery. In addition, the use of laxatives or enemas may also affect the outcomes. Further studies are needed that include these parameters to draw more thorough conclusions related to outcomes.

## Conclusion

Based on our objective criteria of success, the long-term success rate of RS is poor over a median of 74 months.

*Conflict of interest statement*: none declared.

## References

[1] Malouf AJ Norton CS Engel AF Long-term results of overlapping anterior anal-sphincter repair for obstetric trauma. Lancet 2000;355:260–5. 10675072 10.1016/S0140-6736(99)05218-6

[2] Glasgow SC Lowry AC Long-term outcomes of anal sphincter repair for fecal incontinence: a systematic review. Dis Colon Rectum 2012;55:482–90. 22426274 10.1097/DCR.0b013e3182468c22

[3] Tan JJ Chan M Tjandra JJ Evolving therapy for fecal incontinence. Dis Colon Rectum 2007;50:1950–67. 17874167 10.1007/s10350-007-9009-2

[4] Goetz LH Lowry AC Overlapping sphincteroplasty: is it the standard of care? Clin Colon Rectal Surg 2005;18:22–31. 20011336 10.1055/s-2005-864072 PMC2780126

[5] Vaizey CJ Norton C Thornton MJ Long-term results of repeat anterior anal sphincter repair. Dis Colon Rectum 2004;47:858–63. 15129307 10.1007/s10350-003-0112-8

[6] Giordano P Renzi A Efron J Previous sphincter repair does not affect the outcome of repeat repair. Dis Colon Rectum 2002;45:635–40. 12004213 10.1007/s10350-004-6260-7

[7] Pinedo G Vaizey CJ Nicholls RJ Results of repeat anal sphincter repair. Br J Surg 1999;86:66–9. 10027362 10.1046/j.1365-2168.1999.00997.x

[8] Jorge JM Wexner SD Etiology and management of fecal incontinence. Dis Colon Rectum 1993;36:77–97. 8416784 10.1007/BF02050307

[9] Beck DE Wexner SD Fundamentals of Anorectal Surgery. 2nd ed. London: W.B. Saunders, 1998.

[10] Oberwalder M Dinnewitzer A Nogueras JJ Imbrication of the external anal sphincter may yield similar functional results as overlapping repair in selected patients. Colorectal Dis 2008;10:800–4. 18384424 10.1111/j.1463-1318.2008.01484.x

[11] Rothbarth J Bemelman WA Meijerink WJ What is the impact of fecal incontinence on quality of life? Dis Colon Rectum 2001;44:67–71. 11805565 10.1007/BF02234823

[12] Abbas MA Tam MS Chun LJ Radiofrequency treatment for fecal incontinence: is it effective long-term? Dis Colon Rectum 2012;55:605–10. 22513440 10.1097/DCR.0b013e3182415406

[13] Wong MT Meurette G Rodat F Outcome and management of patients in whom sacral nerve stimulation for fecal incontinence failed. Dis Colon Rectum 2011;54:425–32. 21383562 10.1007/DCR.0b013e318200f866

[14] Gallas S Michot F Faucheron JL Predictive factors for successful sacral nerve stimulation in the treatment of faecal incontinence: results of trial stimulation in 200 patients. Colorectal Dis 2011;13:689–96. 20236144 10.1111/j.1463-1318.2010.02260.x

